# Short- and Long-Term Responses to Pulmonary Rehabilitation in 922 Patients with COPD: A Real-World Database Study (2002–2019)

**DOI:** 10.3390/jcm15020793

**Published:** 2026-01-19

**Authors:** Isis Van Raemdonck, Janne van Waterschoot, Yara Vanuytrecht, Dirk Vissers, Thérèse Lapperre, Henrik Hansen

**Affiliations:** 1 Department Rehabilitation Sciences and Physiotherapy, Faculty Medicine and Health Sciences, University of Antwerp, 2610 Antwerp, Belgiumdirk.vissers@uantwerpen.be (D.V.); 2 Department of Pulmonology, University Hospital of Antwerp, 2650 Edegem, Belgium; therese.lapperre@uza.be; 3 Laboratory of Experimental Medicine and Pediatrics, Faculty of Medicine and Health Sciences, University of Antwerp, 2610 Antwerp, Belgium; 4 Respiratory Research Unit, Department of Respiratory Medicine, Copenhagen University Hospital Hvidovre, 2650 Hvidovre, Denmark; 5 Institute of Clinical Medicine, University of Copenhagen, 2200 Copenhagen, Denmark

**Keywords:** COPD, pulmonary rehabilitation, responder analysis, functional capacity, symptoms, real-world dataset

## Abstract

**Background/Objectives:** Pulmonary rehabilitation (PR) is a cornerstone treatment for patients with chronic obstructive pulmonary disease (COPD), yet not all patients achieve clinically meaningful benefits. Evidence on the determinants of short- and long-term responses from real-world settings remains limited. The aim of this study was to quantify response rates to outpatient PR and identify baseline factors associated with achieving minimal clinically important differences (MCIDs) in the walking capacity 6 min walk test [6MWT] or endurance shuttle walk test [ESWT] and patient-reported outcomes (St. George’s Respiratory Questionnaire [SGRQ] or COPD Assessment Test [CAT]) at 10 weeks and 1-year follow-up. **Methods:** In this retrospective cohort study, data from a PR database (2002–2019) at Copenhagen University Hospital Hvidovre were analysed. Patients with COPD and complete data on one functional outcome ([6MWT] or [ESWT]) and one patient-reported outcome ([SGRQ] or [CAT]) were included. Multinomial regression models assessed associations between baseline variables and response categories. **Results:** Among 922 patients, 52% achieved clinically meaningful improvement following PR, and 47% of responders maintained these gains at 1 year. Response rates declined over time. Higher baseline symptom burden (CAT and SGRQ) and walking capacity showed mixed associations with short-term response. Younger age was the most consistent predictor of both short- and long-term responses, while higher baseline FEV_1_ was associated with sustained improvement at 1 year. **Conclusions:** Approximately half of patients with COPD benefit clinically from PR, with sustained improvements in nearly half at 1 year, and response is associated with baseline age, symptom burden, and functional capacity, underscoring the need for a more individualised approach to care.

## 1. Introduction

Pulmonary rehabilitation (PR) is widely recognised as a cornerstone in the treatment of chronic obstructive pulmonary disease (COPD). It has consistently demonstrated effectiveness in improving physical performance, alleviating respiratory symptoms, and enhancing quality of life (QoL) [1]. The European Respiratory Society (ERS) and the American Thoracic Society (ATS) define PR as “a comprehensive intervention based on a thorough patient assessment followed by patient-tailored therapies that include, but are not limited to, exercise training, education, and behaviour change, designed to improve the physical and psychological condition and to promote the long-term adherence to health enhancing behaviours” [2]. In practice, PR typically comprises aerobic and resistance training, patient education, and behavioural interventions, with the broader goals of promoting autonomy, improving overall health status, and supporting sustained healthy lifestyle behaviour [3].

Despite these well-documented benefits of PR, recent evidence reveals highly variable response rates to PR, with 32% to 75% of participants achieving clinically meaningful improvements in exercise capacity and 23% to 92% in health-related QoL [4]. This variability may stem from heterogeneity in study design, sample size, intervention components, and patient characteristics. However, it may also reflect the inconsistent definitions of “response” across studies. Consequently, there is growing concern about the unpredictability of individual response to PR and an increasing need for studies that investigate the determinants of both response and non-response [5,6].

Several factors have been proposed as potential predictors of response to PR. Patients with lower baseline exercise capacity generally exhibit greater improvements in physical performance following PR [7,8,9,10,11]. Similar, poorer baseline lung function has been associated with short-term gains in both exercise tolerance and health-related QoL [5]. Certain physical characteristics, such as higher Body Mass Index (BMI), have also been linked to more favourable outcomes [12]. Moreover, psychosocial factors may influence PR efficacy; for example, strong partner support has been shown to enhance post-rehabilitation functional outcomes [13].

Despite existing research, identifying clear patient characteristics that reliably predict PR response remains challenging. This is largely due to the small sample sizes and narrow inclusion criteria of previous studies, many of which have focused on patients with advanced disease, thereby limiting generalisability [6,14,15]. These limitations underscore the need for updated large-scale investigations.

Accordingly, this study aimed to (a) examine the proportion of patients achieving a minimal clinically important difference (MCID) in physical function and patient-reported outcome measures (PROMs) at 10 weeks and one year post-PR and (b) to identify baseline variables associated with these improvements.

## 2. Methods

### 2.1. Study Design and Setting

This retrospective cohort study was conducted using 18 years of data from an outpatient pulmonary rehabilitation database maintained at Copenhagen University Hospital Hvidovre, Denmark. This study followed the Strengthening the Reporting of Observational Studies in Epidemiology (STROBE) guidelines [16] to ensure transparency and rigour in both analysis and reporting.

### 2.2. Data Sources

The database contains demographic and clinical information from approximately 1600 patients who participated in a 10-week outpatient PR programme at Copenhagen University Hospital Hvidovre.

Data were collected between 2002 and 2019 by rehabilitation staff members who were unblinded to participant information. Assessments were performed at baseline, after 10 weeks of PR, and at one year after PR completion.

### 2.3. Pulmonary Rehabilitation Programme Description

The PR programme was delivered in accordance with the Danish National Guidelines [17,18]. The outpatient PR programme consisted of supervised group-based exercise sessions twice weekly for 10 weeks (20 sessions in total), each lasting 60 min, combined with weekly patient education (60 min). Exercise training included endurance and resistance training delivered according to national and international PR guidelines. Endurance training comprised stationary cycling and functional walking-based exercises, with intensity and typ (continuous or interval) individually prescribed to elicit moderate-to-severe dyspnoea (Borg CR10 score 4–7). Resistance training targeted major upper and lower limb muscle groups at 60–80% of the one-repetition maximum, with systematic progression based on patient performance and tolerance, and training loads were adjusted every 2–4 weeks. Supplemental oxygen during exercise was provided only to patients receiving long-term oxygen therapy (LTOT) and was conservatively titrated (1–2 L·min^−1^ above resting flow). Patients with exertional desaturation performed endurance training in an interval-based format in accordance with guideline recommendations. Following the completion of the 10-week programme, no structured reinforcement or formal maintenance pathway was provided; however, patients were encouraged to continue regular exercise independently. Full details are provided in Supplementary File S1.

### 2.4. Patient Selection

Patient data were included in the analysis if the patient had a confirmed diagnosis of COPD and if data were available at both baseline and post-intervention (10 weeks) for at least one physical outcome measure (endurance shuttle walk test [ESWT] or 6 min walk test [6MWT]) and one patient-reported outcome measure (PROM) (St. George Respiratory Questionnaire [SGRQ] or COPD Assessment Test [CAT]).

### 2.5. Assessments and Variables of Interest

Exercise capacity was assessed using the ESWT or the 6MWT, conducted according to ERS/ATS protocols [19]. The walking test was performed once due to real-world clinical time constraints. Symptom burden and health status were evaluated using the SGRQ or CAT score, completed independently by patients.

Two instrument transitions occurred during the database history. In March 2012, SGRQ was replaced with the CAT, which is shorter and easier for patients to complete while retaining clinically relevant and responsive symptom assessment. In August 2017, the ESWT was replaced with the 6MWT to streamline clinical workflow; both tests offer comparable sensitivity to change following exercise-based rehabilitation. For each patient, the same functional test and PROM were used at baseline, 10 weeks, and 1-year follow-up. MCID-based response was therefore derived from within-instrument change only. Instrument transitions occurred across calendar periods and not within individual patient trajectories.

MCIDs were applied according to the specific outcome available for each patient. Response status was defined as achieving the MCID in at least one physical performance measure and/or one patient-reported outcome measure (PROM) as specified in the *Response Classification*. Baseline values were compared with outcomes at 10 weeks and one year. The baseline variables examined as potential predictors of response included sex [20], age [8,9], FEV_1_ [21], marital status [13], BMI [12,21], ESWT performance [7], 6MWT [6], SGRQ, and CAT [6].

### 2.6. Response Classification

The primary variables used to categorise patients’ responses to PR were as follows: physical outcomes (ESWT or 6MWT) and PROMs (SGRQ or CAT). Patients were categorised as full responders if they achieved the MCID in at least one physical outcome (≥186 s for ESWT [19,22] or ≥30 m for 6MWT [19]) and in at least one PROM (≥4 units for SGRQ [23] or ≥2.5 points for CAT [24,25]). Patients achieving the MCID in only one of the physical measures or PROMs were categorised as responders, while those who did not reach the MCID threshold in either type of measure were classified as non-responders.

### 2.7. Statistics

Descriptive statistics were used to summarise the baseline characteristics of the total cohort and across each response group. Baseline group differences were examined using a one-way analysis of variance (ANOVA) for normally distributed continuous variables, the Kruskal–Wallis test for non-normally distributed continuous variables, and chi-square tests for categorical variables.

A composite variable was created to classify patients as full responders, responders, or non-responders. The proportions of responders were calculated at 10 weeks post-intervention and at one-year follow-up. Changes in response classification between these time points were visualised using a Sankey plot and tested statistically using McNemar’s test, appropriate for paired categorical variables with more than two levels.

To evaluate potential differences in responder rates across the study period (2002–2019), data were grouped into predefined ~10-year time spans (e.g., 2002–2011 and 2012–2019). Time span response rates were compared across these periods using chi-square tests.

To identify baseline variables associated with PR response, logistic regression models were constructed. The likelihood of achieving a response or full response was estimated based on baseline variables, including sex [20], age [8,9], FEV_1_ [21], marital status [13], BMI [12,21], ESWT performance [7], 6MWT [6], SGRQ, and CAT [6]. We limited our analysis to these variables to maintain clarity and focus. Both unadjusted (univariable) and adjusted (multivariable) models were used to account for potential confounding.

All statistical analyses were conducted using IBM SPSS Statistics, version 29.0.2.0. Sankey plots were generated with power-user for Microsoft Excel.

## 3. Results

Following the exclusion of 53 non-COPD patients and 650 patients due to missing data, a total of 922 patients were included in the analysis. The patients excluded due to missing data (*n* = 650) did not differ significantly from the included cohort with respect to sex, age, baseline FEV_1_, ESWT, CAT, or SGRQ scores (61% female; mean ± SD: age 68.8 ± 10.3 years, FEV_1_ 36 ± 12% predicted, ESWT 166 ± 87 s, 6MWD 262 ± 118 m, CAT 19 ± 6 points, SGRQ 55 ± 13 points). Table 1 presents the baseline characteristics of the total cohort, as well as grouped data for the three responder groups at post-intervention and at one-year follow-up. The mean ± SD age of the study population was 69.2 ± 8.7 years with the majority being female, former smokers, and classified as GOLD stage III.

Non-responders were older compared with responders (Table 1). Their endurance time was significantly lower at both 10 weeks and one year follow-up. At 10 weeks, non-responders had a greater 6 min walk distance. There were consistently fewer patient-reported symptoms for non-responders at both time points. At 10 weeks, resting oxygen saturation differed among all groups, with the non-responder group showing the highest percentages (Table 1).

**Figure 1:**
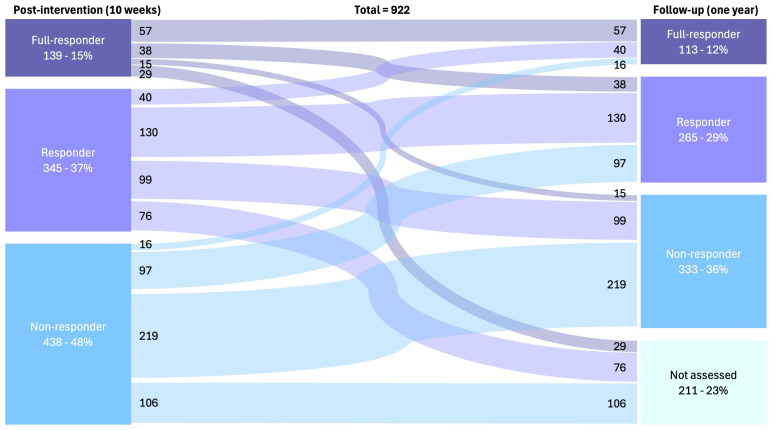
Data are presented as the numbers and percentages of participants classified as full responders (achieving the minimal clinically important difference [MCID] in both a functional test and a patient-reported outcome measure [PROM]), responders (achieving the MCID in either a functional test or a PROM), and non-responders (not achieving the MCID in any outcome).

At 10 weeks, 52% of patients achieved a clinically meaningful response to outpatient PR, of whom 15% were full responders, and 37% were responders. Approximately 47% of these patients maintained these gains at 1-year follow-up (Figure 1). Consequently, 41% of the total cohort was classified as responders or full responders at 1 year. Among patients classified as non-responders at 10 weeks (48%), half remained non-responders at follow-up, while 24% were not re-assessed (Figure 1).

A statistically significant shift in response category distribution was observed between the first (2002–2011) and last (2012–2019) decades, at both 10 weeks (post-intervention) and one-year follow-up (*p* < 0.001 for both) (Table 2). The proportion of full responders was higher in 2002–2011, whereas the proportion of non-responders increased in 2012–2019.

Baseline functional capacity and symptom burden showed statistically significant associations with treatment response (Table 3 and Table 4). A higher baseline ESWT score was consistently associated with both full response and response at 10-week follow-up but not at 1 year. Lower baseline 6 min walk distance was associated with responder status at 10 weeks, but not at 1 year, and was not associated with full response at any time point (Table 3 and Table 4). Patient-reported outcomes demonstrated consistent associations with treatment response. Higher baseline symptom burden, assessed by SGRQ and CAT, was strongly associated with both full response and response at 10 weeks and at 1-year follow-up across all corresponding models (Table 3 and Table 4). Age was consistently associated with response across several outcome models at both short- and long-term follow-ups, while an association with full response was observed at 1 year only in the ESWT- and SGRQ-adjusted models (Table 3 and Table 4).

## 4. Discussion

This study examined short- and long-term response patterns to outpatient pulmonary rehabilitation in a large real-world COPD cohort. Approximately half of patients achieved a clinically meaningful response at 10 weeks, and nearly half of these maintained their gains at 1-year follow-up, although overall response rates declined over time. Importantly, a subset of patients initially classified as non-responders achieved meaningful improvement at 1 year, indicating that early non-response does not preclude later benefit. Across multinomial regression analyses, younger age emerged as the most consistent predictor of both short- and long-term responses. Functional capacity showed heterogeneous associations, with higher baseline endurance capacity (ESWT) and lower baseline walking distance (6MWT) linked to short-term response. In contrast, greater baseline symptom burden, assessed by SGRQ and CAT, was consistently associated with both short- and long-term responses. Other baseline characteristics, including sex, FEV_1_, BMI, and marital status, showed limited or inconsistent associations.

To our knowledge, this is the first study to examine outpatient pulmonary rehabilitation response trajectories over a follow-up period spanning up to 18 years, substantially extending beyond the time frames reported in previous studies. Although half of the patients did not achieve the MCID, a substantial subset demonstrated durable PR benefits, with some even improving further between post-intervention and one year. Notably, approximately one quarter of patients initially classified as non-responders met the response criteria at one year. These late gains may reflect ongoing physiological and behavioural adaptation, but they may also suggest that some post-intervention assessments could be underestimated due to transient factors such as fatigue, comorbidities, fluctuating symptoms, or variable motivation. Day-to-day variability makes it difficult to capture a patient’s “typical” performance at a single time point, leaving room for additional improvement to appear at the one-year follow-up. These patterns highlight heterogeneity in long-term trajectories and reinforce the need for strategies that support physical exercise and activity maintenance once structured PR ends [26,27,28].

Our finding that 52% of outpatients showed a clinically meaningful improvement after pulmonary rehabilitation is comparable to the 54% (“very good responder” + “good responder”) multidimensional responder rate reported by Spruit et al. [11], slightly lower than the 57% reported by Souto-Miranda et al. [29] and 54–64% observed by Vitacca et al. [30] in an in-hospital population. Notably, these comparable response rates were observed despite substantial differences in programme design: Spruit and colleagues used a highly resource-intensive model, whereas the present study reflects a more real-world representative outpatient model typical of Scandinavian settings. Variability in response rates across studies is expected, given differences in response definitions, outcome domains, and population characteristics. The study findings extend the previous observations by examining both the ESWT and 6MWT as physical outcomes and SGRQ and the CAT as PROMs while also drawing on a large and heterogeneous real-world sample. Compared with randomised controlled trials, where stricter selection criteria, blinded assessors, closer supervision, and higher adherence are common, the proportions of responders appear similar [31,32]. This alignment underscores the relationship between efficacy under trial conditions and effectiveness in routine clinical care, highlighting the value of real-world evidence for understanding PR performance in everyday practice.

The lower responder proportion in 2012–2019 likely reflects broader demographic and clinical shifts in the COPD populations treated in an outpatient specialised hospital setting, including older age, greater comorbidity, and more sedentary lifestyles, as well as changing referral patterns [33,34]. These contextual factors align with our regression findings, where younger age and higher baseline exercise capacity and symptom burden predicted more favourable outcomes, underscoring the importance of physiological reserve. This diverges from earlier studies suggesting that lower baseline capacity poses greater benefits [11,30], a discrepancy that may relate to age-related declines in skeletal muscle and cardiovascular capacity. An additional relevant remark is that due to political changes in Denmark during the study period, clinically more stable patients were discharged from hospital care and transitioned to management by their general practitioner. Consequently, only patients with more unstable respiratory symptoms and more comorbidities remained in the hospital-based programme from 2014 and onward. This change plausibly contributed to a shift in the proportion of responders and may partly explain the differences observed across the two-decade intervals.

In our study, a higher baseline ESWT score was associated with a more favourable short-term response. This contrasts with the findings of Vitacca et al. [30], who reported greater improvements in patients with poorer baseline functional status as assessed by the six-minute walk test (6MWT). Their study [30] was conducted on an inpatient COPD population, and they assessed physical and PROM outcomes separately, unlike our analysis using an integrated classification across domains in an outpatient COPD cohort. Secondly, in an inpatient population following a COPD exacerbation, natural recovery post-exacerbation must be considered a mediator to regain in functional capacity and symptom reduction. Thus, the studies offer complementary, rather than directly comparable, insight into responder proportions. For the 6MWT, our findings are consistent with those of Souto-Miranda et al. [29] and Vitacca et al. [30], who reported that patients with a lower baseline 6MWT score were more likely to respond to PR. Although this contrasts with our findings for the ESWT, it supports the general assumption that individuals with lower functional capacity have greater potential for improvement. The apparently opposing associations between the baseline ESWT and 6MWT and response to PR likely reflect fundamental differences in the constructs captured by these walking tests. The ESWT is a constant-load, externally paced test that reflects physiological reserve and endurance capacity; a higher baseline ESWT score may therefore identify patients with greater exertion tolerance to a rapid rise in oxygen uptake and ventilation [19], for example, due to higher walking capacity and less dynamic hyperinflation, enabling them to tolerate higher training intensities and achieve clinically meaningful improvements. In contrast, the 6MWT is self-paced and more strongly influenced by behavioural factors such as pacing strategy and motivation [19]. Patients with lower baseline 6MWT scores may therefore have greater functional limitations but also more “headroom” for improvement and a lower risk of ceiling effects. Together, these findings suggest that the ESWT and 6MWT capture complementary aspects of functional capacity and are associated with response to PR through distinct physiological and behavioural mechanisms.

Higher baseline SGRQ and CAT scores, reflecting higher respiratory symptom burden, were associated with a greater likelihood of response, consistent with previous studies showing that patients with more severe symptoms at baseline derive more benefit from PR [6,11]. This association may reflect a true treatment effect, whereby patients with higher symptom burden have greater potential for clinically meaningful improvement. However, alternative explanations should also be considered. Regression to the mean may partly contribute, as patients assessed during periods of heightened symptoms may show improvement at follow-up independent of intervention effects. In addition, symptom-based measures are inherently sensitive to temporal variation, and the timing of assessment relative to symptom fluctuations may influence observed response. Nevertheless, the consistency of these associations across outcome measures and follow-up time points supports a clinically relevant relationship rather than a purely statistical explanation.

We observed no consistent associations between outpatient pulmonary rehabilitation response and BMI, FEV_1_, marital status, and sex. The lack of association with BMI aligns with previous studies suggesting that BMI is a crude marker of body composition and physical capacity. Indeed, reduced quadriceps strength and greater leg fatigue have been associated with non-response in other cohorts, indicating that direct measures of muscle function may be more informative predictors of response to pulmonary rehabilitation than body composition alone [5,14]. Similarly, although social support has previously been linked to greater gains from pulmonary rehabilitation [13], marital status did not predict response in our study. This likely reflects the fact that marital status is an imperfect proxy for actual social support and that participation in a structured PR may attenuate differences related to external support. With respect to lung function, we found no consistent association. In our ESWT- and SGRQ-adjusted association, we found that higher baseline FEV_1_ was associated with long-term response, which contrasts with earlier work [5,11] reporting that lower FEV_1_ was associated with greater post-PR gains. These previous studies were based on smaller samples and generally comprised patients with less advanced impairments in lung function, which may partly explain the discrepancy. We found no consistent association between sex and outpatient pulmonary rehabilitation response, in line with the findings of Spruit et al. [11]. In contrast, younger age was associated with a more favourable response in our cohort, whereas no age effect was observed in some previous studies. However, the existing literature indicates that responders are often numerically, but not statistically significantly, younger, with absolute age differences across studies, including ours, typically in the range of 1–2 years. Such small differences are unlikely to be clinically meaningful, suggesting that age should be interpreted as a contextual modifier rather than a decisive predictor of pulmonary rehabilitation response.

A key distinction between cohorts is that Spruit et al. included both inpatients and outpatients, with inpatients demonstrating a stronger response [8,11,29,35]. This may reflect a combination of natural recovery and the intensive, around-the-clock rehabilitation intervention applied in their study [11], as well as fewer external barriers such as transportation and scheduling.

The generalisability of our findings is supported by this large, heterogeneous outpatient cohort representative of routine clinical practice, predominantly comprising patients with advanced COPD and collected over an 18-year period. The combined use of physical and patient-reported outcomes captures the multidimensional effects of PR and enhances the clinical relevance of the response classification. Nevertheless, caution is warranted when extrapolating these results to patients with milder stages of COPD or to healthcare systems with substantially different PR structures.

From a clinical perspective, the observed heterogeneity in response underscores the need for individualised expectations and may inform the tailoring of PR programmes or supportive strategies based on baseline characteristics, particularly age, functional status, and prior PR experience. The additional consideration of outcome domains not captured by our standard outcomes, such as frailty, fatigue, or comorbidity burden, may help contextualise non-response. Important knowledge gaps remain, including mechanisms underlying non-response and loss or delayed gains over time, which warrant further investigation alongside the consideration of temporal shifts in patient profiles.

This study has several strengths. It is based on a large, real-world cohort with long-term follow-up, supporting the external validity and clinical relevance of the findings. The integration of objective measures of functional capacity and validated patient-reported outcomes enables a comprehensive assessment of response to pulmonary rehabilitation. In addition, the use of a consistent analytical approach over an extended time period allows for the evaluation of temporal trends in treatment response under routine clinical conditions. Some limitations should be acknowledged. Response classification was based on established MCIDs, and alternative thresholds may have yielded different responder distributions. Although regression analyses included prespecified clinically relevant baseline variables, residual confounding from unmeasured factors cannot be excluded, as expected in observational studies. Attrition at 1-year follow-up may have influenced the estimates of sustained response; however, this reflects real-world clinical practice, and no assumptions were made regarding outcomes among non-assessed patients. The cohort predominantly comprised patients with severe to very severe COPD, which may limit generalisability to individuals with milder disease, as lower GOLD stages were underrepresented. In addition, detailed and standardised comorbidity indices (e.g., Charlson or COTE) were not available in the database, limiting our ability to assess the impact of comorbidities on rehabilitation outcomes. Finally, the retrospective design and unblinded nature of data collection spanning several decades imply that rehabilitation practices and outcome assessments likely evolved over time, introducing heterogeneity that reflects real-world care but may also contribute to outcome variability.

## 5. Conclusions

Following outpatient PR, 52% of patients with COPD demonstrated clinically meaningful improvements, 15% as full responders and 37% as responders. Approximately 47% of patients maintained these gains at one year. Response rates declined over time, with fewer responders in the most recent decade. Younger age was the most consistent predictor of both short- and long-term responses. Baseline walking capacity showed mixed associations and was only related to short-term response, whereas a higher baseline symptom burden (CAT and SGRQ) consistently predicted both short- and long-term outcomes.

## Figures and Tables

**Table 1:** Baseline patient characteristics for different responder subgroups and total population.

Variables (*n*)	Baseline Characteristics Total Population (*n* = 922)	Response After 10 Weeks			Response After One Year		
		Full Response (*n*= 139)	Response (*n* = 345)	Non-Response (*n* = 438)	Full Response (*n* = 113)	Response (*n* = 265)	Non-Response (*n* = 333)
Age, yrs (920)	69.2 ± 8.7	67.9 ± 8.9 ¶	68.8 ± 8.9	69.9 ± 8.4 *	66.6 ± 9.3 ¶	68.6 ± 8.9	70.1 ± 8.0 *
Female sex, *n* (%) (922)	577 (62.6)	81 (58.3)	226 (65.5)	270 (61.6)	70 (61.9)	159 (60.0)	214 (64.3)
FEV_1_, %pred (909) FEV_1_/FVC, % (885)	35.5 ± 14.1 47.0 ± 13.9	33.9 ± 12.2 47.2 ± 13.0	36.2 ± 16.7 47.4 ± 14.7	35.5 ± 12.4 46.6 ± 13.5	35.4 ± 14.2 47.7 ± 13.4	35.9 ± 13.2 48.3 ± 13.7 ¶	34.3 ± 12.1 45.1 ± 14.3 #
GOLD I/II/III/IV, % (908)	0.4/11.5/51.4/35.1	0/10.8/45.3/43.2	0.6/12.8/50.4/35.1	0.5/10.7/54.1/32.6	1.8/10.6/46.9/39.8	0.4/15.1/49.1/34.3	0.3/8.7/52.3/37.5
Smoking status, % (912) Never/former/current Pack-year history, yrs (861)	2.7/76.9/19.2 41.4 ± 25.3	3.6/69.8/24.5 40.9 ± 19.3	1.7/78.3/19.1 40.3 ± 18.9	3.2/78.1/17.6 42.3 ± 30.9	2.7/77.9/17.7 40.3 ± 21.0	1.1/79.2/18.5 41.0 ± 21.1	2.4/79.3/18.0 41.9 ± 30.5
LTOT, *n* (%) (919)	62 (6.7)	12 (8.6)	17 (4.9)	33 (7.5)	8 (7.1)	16 (6.0)	23 (6.9)
BMI, kg/m^2^ (915)	25.6 ± 6.0	25.1 ± 5.9	25.6 ± 6.3	25.8 ± 5.8	25.4 ± 5.9	25.8 ± 6.0	25.4 ± 5.9
Cardiac disease, *n* (%) (918) Musculoskeletal disease, *n* (%) (918)	332 (36.0) 233 (25.3)	51 (36.7) 44 (31.7)	121 (35.1) 84 (24.3)	160 (36.5) 105 (24.0)	40 (35.4) 32 (28.3)	99 (37.4) 62 (23.4)	116 (34.8) 75 (22.5)
Number of hospital admissions within the last year, *n* (904)	0.8 ± 1.5	1.0 ± 1.6	0.8 ± 1.5	0.8 ± 1.4	1.0 ± 1.5	0.7 ± 1.5	0.8 ± 1.4
Number of hospital days within the last year, *n* (874)	4.7 ± 10.0	5.3 ± 9.9	4.7 ± 10.6	4.6 ± 9.6	4.5 ± 8.1	4.4 ± 10.3	5.0 ± 10.7
Current medication, *n* (%) SABA (913) LABA (914) LAMA (913) LABA + LAMA(922) LABA + ICS (922) LABA + LAMA + ICS (912) ICS (913) Oral steroids (913)	774 (83.9) 438 (47.5) 705 (76.5) 339 (36.8) 366 (39.7) 296 (32.1) 752 (81.6) 61 (6.6)	113 (81.3) 75 (54.0) 104 (74.8) 61 (43.9) 66 (47.5) 55 (39.6) 110 (79.1) 11 (7.9)	291 (84.3) 162 (47.0) 258 (74.8) 117 (33.9) 139 (40.3) 106 (30.7) 289 (83.8) 22 (6.4)	370 (84.5) 201 (45.9) 343 (78.3) 161 (36.8) 161 (36.8) 135 (30.8) 353 (80.6) 28 (6.4)	92 (81.4) 53 (46.9) 88 (77.9) 43 (38.1) 48 (42.5) 39 (34.5) 97 (85.8) 10 (8.8)	223 (84.2) 127 (47.9) 200 (75.5) 95 (35.8) 104 (39.2) 85 (32.1) 212 (80.0) 17 (6.4)	287 (86.2) 154 (46.2) 263 (79.0) 120 (36.0) 133 (39.9) 105 (31.5) 281 (84.4) 24 (7.2)
Marital status, *n* (%) (917) Married/living with partner Living alone Nursing home	440 (47.7) 476 (51.6) 1 (0.1)	75 (54.0) 63 (45.3) 0 (0)	154 (44.6) 190 (55.1) 1 (0.3)	211 (48.2) 223 (50.9) 1 (0.1)	58 (51.3) 55 (48.7) 0 (0.0)	127 (47.9) 138 (52.1) 0 (0.0)	153 (45.9) 177 (53.2) 1 (0.3)
Education years, *n* (898)	9.0 ± 2.6	9.4 ± 2.6	8.9 ± 2.7	8.8 ± 2.3	9.5 ± 3.0	9.0 ± 2.4	8.7 ± 2.5
Working status, *n* (%) (906) Working Retired Disability pension Vocational work placement	30 (3.3) 288 (31.2) 305 (33.1) 283 (30.79)	6 (4.3) 47 (33.8) 49 (35.3) 35 (25.2)	11 (3.2) 112 (32.5) 118 (34.2) 101 (29.3)	13 (3.0) 129 (29.5) 138 (31.5) 147 (33.6)	6 (5.3) 33 (29.2) 39 (34.5) 33 (29.2)	7 (2.6) 98 (37.0) 88 (33.2) 71 (26.8)	11 (3.3) 106 (31.8) 105 (31.5) 105 (31.5)
Borg CR-10 score at rest (920)	0.6 ± 1.0	0.7 ± 1.1	0.6 ± 1.1	0.6 ± 1.0	0.6 ± 1.0	0.6 ± 1.1	0.6 ± 1.1
HR at rest (920)	82.3 ± 14.0	82.9 ± 13.7	81.9 ± 15.0	82.3 ± 13.3	84.0 ± 15.9	83.0 ± 13.0	81.2 ± 14.3
Saturation at rest (920)	95.0 ± 26.5	93.9 ± 2.1 # ¶	94.3 ± 2.2 * ¶	95.9 ± 38.4 * #	94.3 ± 2.1	97.2 ± 49.3	94.0 ± 2.1
MRC score, median (IQR) (921)	4.5 (1–5)	3.5 (2–5)	3.5 (1–5)	4.5 (1–5)	3.5 (2–5)	3.5 (2–5)	4.5 (2–5)
Endurance SWT Walking speed, km/h (910) Time, seconds (861) End HR (857) End saturation (%) (858) End Borg CR-10 score (860)	3.3 ± 1.1 172.5 ± 93.4 107.7 ± 14.4 88.3 ± 5.5 4.8 ± 1.6	3.2 ± 0.9 180.7 ± 92.8 107.8 ± 12.6 88.3 ± 5.4 4.8 ± 1.5	3.3 ± 1.2 180.9 ± 100.8 ¶ 108.3 ± 15.3 88.6 ± 5.4 4.9 ± 1.6	3.3 ± 1.0 163.2 ± 86.5 # 107.3 ± 14.1 88.0 ± 5.6 4.8 ± 1.6	3.1 ± 0.7 186.1 ± 84.7 ¶ 108.3 ± 14.5 89.2 ± 4.8 ¶ 4.6 ± 1.4	3.3 ± 0.8 175.6 ± 88.7 108.6 ± 13.3 88.1 ± 5.9 4.9 ± 1.6	3.2 ± 1.2 171.1 ± 105.2 * 108.0 ± 14.9 87.6 ± 5.4 * 4.9 ± 1.6
6MWT 6MWD, metres (62) 6MWD, %pred (62) End saturation (%) (60) Numbers of pauses during test (60) End Borg CR-10 score (60)	299.1 ± 87.2 50 ± 15 88.3 ± 5.0 1.1 ± 1.4 5.1 ± 1.7	259.3 ± 64.8 ¶ 44 ± 10 ¶ 89.8 ± 4.2 1.5 ± 1.4 5.2 ± 1.7	272.2 ± 98.8 ¶ 48 ± 19 89.6 ± 4.0 1.1 ± 1.4 5.4 ± 1.8	330.6 ± 76.1 * # 55 ± 13 * 86.8 ± 5.5 0.9 ± 1.5 4.9 ± 1.7	268.3 ± 58.9 42 ± 11 ¶ 93.0 ± 6.1 1.0 ± 1.0 4.3 ± 0.6	272.6 ± 108.5 47 ± 17 89.9 ± 4.3 1.1 ± 1.1 5.1 ± 2.1	306.5 ± 93.6 53 ± 14 * 86.1 ± 5.1 0.6 ± 1.0 5.6 ± 1.5
SGRQ total score (652) Symptom score (652) Activity score (652) Impact score (652)	54.2 ± 13.3 60.7 ± 19.5 75.8 ± 14.5 40.1 ± 15.8	58.9 ± 12.9 ¶ 66.6 ± 17.9# ¶ 77.8 ± 15.1 ¶ 46.3 ± 5.7 # ¶	55.8 ± 13.3 ¶ 62.0 ± 19.3 * ¶ 77.4 ± 14.9 ¶ 41.8 ± 15.4 * ¶	51.0 ± 12.7 * # 57.2 ± 19.7 * # 73.8 ± 13.8 * # 36.3 ± 15.3 * #	56.7 ± 13.4 ¶ 64.2 ± 18.7 ¶ 77.8 ± 15.1 ¶ 42.5 ± 16.7 ¶	56.2 ± 12.8 ¶ 63.5 ± 17.7 ¶ 77.4 ± 13.7 ¶ 42.3 ± 15.0 ¶	51.7 ± 12.6 * # 58.2 ± 19.7 * # 74.0 ± 14.6 * # 37.2 ± 15.2 * #
CAT score (274)	18.7 ± 6.7	23.2 ± 5.3 # ¶	19.5 ± 6.1 * ¶	17.4 ± 6.8 * #	22.1 ± 5.1 ¶	20.1 ± 6.1 ¶	15.7 ± 6.3 * #

Data are presented as mean ± SD, unless otherwise indicated. * *p* < 0.05 versus full responders, # *p* < 0.05 versus responders, ¶ *p* < 0.05 versus non-responders; one-way ANOVA for normally distributed continuous variables, Kruskal–Wallis for non-normally distributed continuous variables, and lastly chi-squared for categorical variables. Abbreviations: %pred, percentage of the predicted value; FEV1, forced expiratory volume in 1 s; FVC, forced vital capacity; FEV1/FVC, Tiffeneau index; GOLD, Global Initiative for Obstructive Lung Disease; LTOT, long-term oxygen therapy; BMI, body mass index; SABA, short-acting beta-2 antagonist; LABA, long-acting beta-2 antagonist; LAMA, long-acting muscarinic antagonist; ICS, inhaled corticosteroid; IQR, interquartile range; m, metre; MRC, Medical Research Council Dyspnea Scale; End Borg CR-10, rating of dyspnoea; HR, heart rate; sec, seconds; SWT, shuttle walk test; 6MWT, 6-minute walk test; 6MWD, 6-minute walk distance; SGRQ, St. George’s Respiratory Questionnaire; CAT, COPD Assessment Test.

**Table 2:** Longitudinal assessment of responder status across 10-year intervals from baseline to 10 weeks and 1-year follow-up.

	Response from Baseline to 10 Weeks (Post-Intervention)		Response from Baseline to One-Year Follow-Up	
	Interval 2002–2011 (*n* = 647)	Interval 2012–2019 (*n* = 275)	Interval 2002–2011 (*n* = 647)	Interval 2012–2019 (*n* = 275)
Full Responder, *n* (%)	108 (16.7) *	31 (11.3)	98 (17.5) *	16 (8.2)
Responder, *n* (%)	253 (39.1) *	92 (33.5)	198 (35.4)	81 (41.5)
Non-Responder, *n* (%)	286 (44.2) *	152 (55.3)	264 (47.1) *	98 (50.3)

Data are presented as numbers (*n*) and percentages (%). Statistically significant difference between decades is denoted as * *p* < 0.05. Participants are classified as full responders (achieving minimal clinically important difference [MCID] in both functional test and patient-reported outcome measure [PROM]), responders (achieving MCID in either functional test or PROM), and non-responders (not achieving MCID in any outcome).

**Table 3:** Adjusted multinomial regression analysis of factors associated with full and partial responses at 10 weeks.

		Full Responders		Responders	
	Variable	Odds Ratio (Confidence Interval)	*p*-Value	Odds Ratio (Confidence Interval)	*p*-Value
10-week follow-up (adjusted for ESWT) (*n* = 701)	ESWT	1.003 (1.000–1.005)	0.020 *	1.002 (1.000–1.004)	0.045 *
	Sex	0.915 (0.566–1.479)	0.717	0.941 (0.656–1.349)	0.741
	Age	0.976 (0.951–1.002)	0.069	0.980 (0.961–0.999)	0.044 *
	FEV_1_	0.994 (0.975–1.014)	0.561	1.004 (0.990–1.018)	0.605
	BMI	0.970 (0.931–1.011)	0.150	0.988 (0.960–1.016)	0.560
	Marital status	0.790 (0.494–1.261)	0.322	1.273 (0.900–1.800)	0.173
10-week follow-up (adjusted for 6MWT) (*n* = 39)	6MWT	0.994 (0.983–1.006)	0.326	0.989 (0.980–0.999)	0.024 *
	Sex	0.148 (0.011–2.075)	0.156	0.517 (0.095–2.806)	0.444
	Age	1.034 (0.865–1.236)	0.711	1.001 (0.874–1.148)	0.984
	FEV_1_	1.025 (0.920–1.142)	0.651	1.009 (0.931–1.095)	0.820
	BMI	0.991 (0.802–1.223)	0.930	1.092 (0.951–1.254)	0.214
	Marital status	1.210 (0.117–12.471)	0.873	0.472 (0.086–2.597)	0.388
10-week follow-up (adjusted for SGRQ) (*n* = 552)	SGRQ	1.040 (1.020–1.061)	<0.001 *	1.026 (1.011–1.041)	<0.001 *
	Sex	0.954 (0.556–1.639)	0.865	0.842 (0.556–1.274)	0.416
	Age	0.980 (0.952–1.010)	0.183	0.972 (0.950–0.994)	0.014 *
	FEV_1_	0.998 (0.977–1.019)	0.819	1.010 (0.995–1.025)	0.194
	BMI	0.970 (0.926–1.015)	0.187	0.987 (0.955–1.021)	0.456
	Marital status	0.576 (0.342–0.970)	0.038 *	1.084 (0.728–1.616)	0.691
10-week follow-up (adjusted for CAT) (*n* = 189)	CAT	1.191 (1.076–1.319)	<0.001 *	1.042 (0.988–1.100)	0.131
	Sex	0.300 (0.093–0.967)	0.044 *	0.951 (0.476–1.900)	0.888
	Age	1.019 (0.953–1.090)	0.583	1.034 (0.992–1.079)	0.115
	FEV_1_	1.023 (0.969–1.080)	0.403	1.002 (0.969–1.036)	0.897
	BMI	0.916 (0.823–1.018)	0.101	0.994 (0.943–1.048)	0.830
	Marital status	1.688 (0.540–5.272)	0.368	1.235 (0.626–2.439)	0.542

Data are presented as the OR (95% CI). Statistically significant denoted *. The adjusted multinominal logistic regression analysis included the presented covariates sex, age, FEV_1_, BMI (as a continuous variable), and marital status. Reference category: Non-responders. Abbreviation: FEV_1_, Forced Expiratory Volume in 1 s; BMI, Body Mass Index; ESWT, Endurance Shuttle Walk Test; 6MWT, 6-Minute Walk Test; SGRQ, St. George’s Respiratory Questionnaire; CAT, COPD Assessment Test.

**Table 4:** Adjusted multinomial regression analysis of factors associated with full and partial responses at 1-year follow-up.

		Full Responders		Responders	
	Variable	Odds Ratio (Confidence Interval)	*p*-Value	Odds Ratio (Confidence Interval)	*p*-Value
One-year follow-up (adjusted for ESWT) (*n* = 701)	ESWT	1.001 (0.999–1.003)	0.240	1.000 (0.999–1.002)	0.682
	Sex	0.928 (0.577–1.490)	0.756	0.870 (0.607–1.247)	0.449
	Age	0.953 (0.929–0.977)	0.016 *	0.974 (0.955–0.994)	0.010 *
	FEV_1_	1.017 (0.998–1.035)	0.078	1.015 (1.001–1.029)	0.039 *
	BMI	0.991 (0.954–1.030)	0.658	0.996 (0.967–1.025)	0.767
	Marital status	0.816 (0.516–1.291)	0.386	1.041 (0.736–1.472)	0.820
One-year follow-up (adjusted for 6MWT) (*n* = 39)	6MWT	0.991 (0.977–1.005)	0.220	0.990 (0.981–1.000)	0.055
	Sex	0.086 (0.003–2.217)	0.139	0.048 (0.005–0.501)	0.011 *
	Age	0.877 (0.667–1.153)	0.347	1.059 (0.904–1.240)	0.479
	FEV_1_	1.019 (0.876–1.185)	0.808	1.062 (0.969–1.164)	0.200
	BMI	0.945 (0.746–1.197)	0.638	1.069 (0.915–1.249)	0.399
	Marital status	5.793 (0.279–120.059)	0.256	4.949 (0.550–44.509)	0.154
One-year follow-up (adjusted for SGRQ) (*n* = 552)	SGRQ	1.028 (1.009–1.048)	0.003 *	1.027 (1.012–1.043)	<0.001 *
	Sex	1.002 (0.594–1.692)	0.993	0.874 (0.574–1.331)	0.530
	Age	0.955 (0.928–0.982)	0.001 *	0.975 (0.953–0.998)	0.036 *
	FEV_1_	1.021 (1.001–1.040)	0.038 *	1.019 (1.003–1.035)	0.018 *
	BMI	0.988 (0.948–1.031)	0.583	0.986 (0.953–1.021)	0.437
	Marital status	0.687 (0.416–1.134)	0.142	0.907 (0.605–1.357)	0.634
One-year follow-up (adjusted for CAT) (*n* = 189)	CAT	1.169 (1.055–1.295)	0.003 *	1.113 (1.053–1.177)	<0.001 *
	Sex	0.322 (0.098–1.064)	0.063	0.567 (0.287–1.119)	0.102
	Age	0.987 (0.923–1.057)	0.716	1.019 (0.979–1.062)	0.354
	FEV_1_	1.012 (0.955–1.073)	0.678	1.011 (0.978–1.045)	0.526
	BMI	0.952 (0.860–1.054)	0.343	1.007 (0.956–1.061)	0.787
	Marital status	1.500 (0.457–4.923)	0.503	1.454 (0.741–2.853)	0.277

Data are presented as the OR (95% CI). Statistically significant denoted *. The adjusted multinominal logistic regression analysis included the presented covariates sex, age, FEV_1_, BMI (as a continuous variable), and marital status. Reference category: Non-responders. Abbreviation: FEV_1_, Forced Expiratory Volume in 1 s; BMI, Body Mass Index; ESWT, Endurance Shuttle Walk Test; 6MWT, 6-Minute Walk Test; SGRQ, St. George’s Respiratory Questionnaire; CAT, COPD Assessment Test.

## Data Availability

No new data were created or analyzed in this study.

## Supplementary Materials

The following supporting information can be downloaded at: https://www.mdpi.com/article/10.3390/jcm15020793/s1.
