# Supplementary material for: Short- and Long-Term Responses to Pulmonary Rehabilitation in 922 Patients with COPD: A Real-World Database Study (2002–2019)
Source: J Clin Med. 2026 Jan 19;15(2):793. doi: 10.3390/jcm15020793 (PMC12841861; doi:10.3390/jcm15020793)
Supplement: Supplementary file 1 [file jcm-15-00793-s001.zip › jcm-4099132-supplementary.pdf]

SUPPLEMENT 1

Conventional outpatient pulmonary rehabilitation programme (PR)

at Copenhagen University Hospital Amager and Hvidovre, Denmark

Patients with COPD underwent an out-patient and supervised standard pulmonary rehabilitation program (PR) in groups of 6–12 patients, following the Danish Health Authority's National Clinical Guideline and the Regional Guidelines[1–4]. The out-patient PR program includes individually tailored physical exercise and patient education. Exercise sessions last 60 minutes twice weekly (a weekly exercise volume of 120 minutes) for 10 weeks supervised by two skilled physiotherapists with at least two years of experience with PR. The exercises in the PR exercise program are well-documented endurance and resistance exercises[5], presented in Table S1. The time volume allocated for endurance and resistance training modalities are equal. Endurance training always includes 15 minutes of stationary cycling, performed in intervals or as continuous cycling, depending on patient preference, desaturation, hip/knee/back pain and other comorbidities. Another 5–15 minutes of endurance training are performed as functional exercises in, for example, paced walking, stairclimbing or circuit training. Intensities are set to reach dyspnea corresponding to a Borg score of CR10, 4–7, depending on whether exercises were performed continuously or as intervals.

Resistance training involves large muscle groups with 50/50 % of exercises for upper and lower extremities, respectively [5–12]. The volume, intensity and content specified is in accordance with both national, regional and international exercise recommendations to assure the appropriate dosage of exercise and intensity [2,3,5,6,13–15]. The exercises are executed in two to three sets of 8 to 20 repetitions (corresponding to 60–80% of 1RM) to achieve peripheral muscle fatigue and muscle strengthening (Table S1). A pause of 1–2 minutes between each

set is mandatory. Exercises are conducted using three strength training machines (leg press, knee extension and chest press or pulldown) supplemented with dumbbells, elastic bands, and weight cuffs. Resistance values are readjusted every 2nd to 4th week and depend on training adherence, repetition count, patient feedback and motivation[16,17]. A familiarization phase to adapt to exercising, adjust and optimize load and avoid demotivation and musculoskeletal overload injuries span 2–4 sessions for each patient. A patient education session of 60 minutes takes place once a week (Mondays) following the exercise session and is led by a trained respiratory nurse with at least two years of PR experience. A chest physician, a physiotherapist and a dietician separately lead one of the ten sessions, respectively, during the education period. The total number of patient education sessions are 10. Topics cover the education program and the didactics are presented in Table S2 and are disseminated as a combination of dialog, reflection exercises and practical exercises[3,18] (Table S2).

Table S1. Exercise content conventional out-patient pulmonary rehabilitation			
**Exercise type**	**Exercises**	**Intensity**	**Progression**
Warm-up<br>(duration 5-10min)	Sitting or standing:<br>-heel uprisings<br>(uni- or bilateral)<br>- knee extension<br>- rear deltoid row<br>- chest press movement<br>- vertical shoulder press<br>(uni- or bilateral)<br><br>Standing:<br>-walking various<br>- leg curl<br>- leg swing<br>- squats	Non-specific intensity<br><br>Purpose:<br>-increase body temperature<br>- cardiorespiratory warm-up<br>-muscle and tendon tissue warm-up	none
Endurance training<br>(duration 20-30min)	-Walking or<br>-Cycle or<br>- Treadmill or<br>- Circuit training or<br>- Activity games	Borg CR-10 dyspnea 4-7<br><br>Exercises performed in intervals or continuously	Every 2nd to 4th week load adjustment individualized
Resistance training<br>Duration 20-30min)	Machine:<br>-leg press<br>-knee extension<br>Pull down and/or<br>chest press (vertical)<br><br>Other equipment for strength circuit training<br>elastic band<br>dumbbells<br>weight cuff	50-80% of 1RM corresponding to 8-20 repetitions<br>2-3 sets	Every 2nd to 4th week load adjustment individualized<br>(repetition counting by supervisor)
Cool-down<br>(duration 5-10min)	Breathing exercises<br>Pursed lip breathing<br>Relaxation exercises<br>Yoga exercises	Non-specific intensity	Non-specific

Health professional responsible: Physiotherapist
The monitoring of intensity may vary, but it is expected that the program uses either objective (pulse or Watt monitoring) or subjective (CR Borg scale for dyspnea) measurements for intensity monitoring.
Resistance training will be evaluated for progression by counting the maximum repetitions and estimating a new optional weight/resistance within 8-20 repetitions.
Workout logs from every training session are recommended to be registered by the authorization law.

Table S2. Patient education topics control group—conventional pulmonary rehabilitation	
**Topics/themes**	**Communication/learning form**
• COPD and the treatment<br>• The importance of smoking cessation<br>• The importance of daily activity and exercise<br>• The importance of nutrition<br>• Medication and use of devices and inhalation techniques<br>• Early signs of exacerbation and action plan<br>• Use of nebulizer apparatus and oxygen apparatus.<br><br>Individual smoking cessation and dietary advice will be offered to the individual COPD patient if assessed relevant.	Topics are promoted as a combination of<br>• Information<br>• Dialog<br>• Reflection exercises<br>• Practical exercises<br>• Focusing on increasing the individual's self-competence<br>• Networking and exchange of experience.
Health professional responsible: Respiratory nurse	

References

1. Danish National board of Health. *National Klinisk Retningslinje for Rehabilitering Af Patienter Med KOL.*; 2018. Accessed October 2, 2018. https://www.sst.dk/da/udgivelser/2018/~/media/AD2FF426014943D983E0D7B937B356B9.ashx

2. Mølsted S, Dall CH, Hansen H, Beyer N. *Anbefalinger Til Superviseret Fysisk Traening Af Mennesker Med Type 2-Diabetes, KOL Og Hjerte-Kar-Sygdom.*; 2012. Accessed February 12, 2017. https://www.regionh.dk/til-fagfolk/Sundhed/Tvaersektorielt-samarbejde/kronisk-sygdom/PublishingImages/Sider/Rehabilitering/2206717238_RapportRHLauraJennifer-Munkø_low.pdf

3. Capital Region D. *Forløbsprogram for KOL Hospitaler, Almen Praksis Og Kommunerne i Region Hovedstaden.*; 2015. Accessed February 12, 2017. https://www.regionh.dk/Sundhedsaftale/bilag-og-download/Documents/RH_Program_KOL_rev_2015.pdf

4. Martinez G, Hansen H, Johannesen G, Godtfredsen N. *Dansk Lungemedicinsk Selskab.* Vol 5.; 2024. https://lungemedicin.dk/wp-content/uploads/2021/05/DLS-Lungerehabilitering_feb2023.pdf

5. Spruit MA, Singh SJ, Garvey C, et al. An official American thoracic society/European respiratory society statement: Key concepts and advances in pulmonary rehabilitation. *Am J Respir Crit Care Med.* 2013;188(8). doi:10.1164/rccm.201309-1634ST

6. Uk JB, Woodcock A, Knight A, et al. BTS Guideline on Pulmonary Rehabilitation in Adults. *An Int J Respir Med.* 2013;68(2):ii1-31. doi:10.1136/thoraxjnl-2013-203808

7. McFarland C, Willson D, Sloan J, Coultas D. A randomized trial comparing 2 types of in-home rehabilitation for chronic obstructive pulmonary disease: a pilot study. *J Geriatr Phys Ther.* 2012;35(3):132-139. doi:10.1519/JPT.0b013e31824145f5

8. Nyberg A, Lindström B, Wadell K. Assessing the effect of high-repetitive single limb exercises

(HRSLE) on exercise capacity and quality of life in patients with chronic obstructive pulmonary disease (COPD): study protocol for randomized controlled trial. *Trials*. 2012;13(1):114. doi:10.1186/1745-6215-13-114

9. Janaudis-Ferreira T, Hill K, Goldstein RS, et al. Resistance Arm Training in Patients With COPD. *Chest*. 2011;139(1):151-158. doi:10.1378/chest.10-1292

10. Costi S, Crisafulli E, Degli Antoni F, Beneventi C, Fabbri LM, Clini EM. Effects of Unsupported Upper Extremity Exercise Training in Patients With COPD: A Randomized Clinical Trial. *Chest*. 2009;136(2):387-395. doi:10.1378/chest.09-0165

11. Gosker HR, Lencer NHMK, Franssen FME, van der Vusse GJ, Wouters EFM, Schols AMWJ. Striking similarities in systemic factors contributing to decreased exercise capacity in patients with severe chronic heart failure or COPD. *Chest*. 2003;123(5):1416-1424. http://www.ncbi.nlm.nih.gov/pubmed/12740256

12. Man WDC, Soliman MGG, Gearing J, et al. Symptoms and quadriceps fatigability after walking and cycling in chronic obstructive pulmonary disease. *Am J Respir Crit Care Med*. 2003;168(5):562-567. doi:10.1164/rccm.200302-162OC

13. National Board of Health. *National Klinisk Retningslinje for Rehabilitering Af Patienter Med KOL.*; 2014. Accessed February 12, 2017. https://www.sst.dk/da/nyheder/2014/~/media/C0B92E9C3BEA4B28A3294D70288EC535.ashx

14. Pedersen BK, Saltin B. Exercise as medicine - evidence for prescribing exercise as therapy in 26 different chronic diseases. *Scand J Med Sci Sports*. 2015;25 Suppl 3:1-72. doi:10.1111/sms.12581

15. Troosters T, Casaburi R, Gosselink R, Decramer M. Pulmonary rehabilitation in chronic obstructive pulmonary disease. *Am J Respir Crit Care Med*. 2005;172(1):19-38. doi:10.1164/rccm.200408-1109SO

16. Campos GER, Luecke TJ, Wendeln HK, et al. Muscular adaptations in response to three different resistance-training regimens: Specificity of repetition maximum training zones. *Eur J Appl Physiol*. 2002;88(1-2):50-60. doi:10.1007/s00421-002-0681-6

17. Linda S Pescatello; Ross Arena; Deborah Riebe; Paul D Thompson; American College of Sports Medicine. *ACSM's Guidelines for Exercise Testing and Prescription*. 9th ed. (Lippincott Williams & Wilkins.;, ed.). Lippincott Williams & Wilkins.; 2014.

18. Rice KL, Dewan N, Bloomfield HE, et al. Disease management program for chronic obstructive pulmonary disease a randomized controlled trial. *Am J Respir Crit Care Med*. 2010;182(7):890-896. doi:10.1164/rccm.200910-1579OC
